# Gender Differences in the Prevalence of Parkinson's Disease

**DOI:** 10.1002/mdc3.13584

**Published:** 2022-11-14

**Authors:** Alexandra Zirra, Shilpa C. Rao, Jonathan Bestwick, Rajasumi Rajalingam, Connie Marras, Cornelis Blauwendraat, Ignacio F. Mata, Alastair J. Noyce

**Affiliations:** ^1^ Preventive Neurology Unit Wolfson Institute of Population Health, Queen Mary University of London London United Kingdom; ^2^ Genomic Medicine Institute Lerner Research Institute, Cleveland Clinic Foundation Cleveland Ohio USA; ^3^ Department of Molecular Medicine Case Western Reserve University Cleveland Ohio USA; ^4^ University Health Network University of Toronto Toronto Ontario Canada; ^5^ Laboratory of Neurogenetics, National Institute of Aging National Institutes of Health Bethesda Maryland USA; ^6^ Department of Clinical and Movement Neurosciences UCL Institute of Neurology London United Kingdom

**Keywords:** Parkinson's disease, meta‐analysis, systematic review, prevalence, gender differences.

## Abstract

**Background:**

Parkinson's disease (PD) affects males more than females. The reasons for the gender differences in PD prevalence remain unclear.

**Objective:**

The objective of this systematic review and meta‐analysis was to update the overall male/female prevalence ratios (OPR).

**Methods:**

We updated previous work by searching MEDLINE, SCOPUS, and OVID for articles reporting PD prevalence for both genders between 2011 and 2021. We calculated OPRs and investigated heterogeneity in effect estimates.

**Results:**

We included 19 new articles and 13 articles from a previously published meta‐analysis. The OPR was 1.18, 95% CI, [1.03, 1.36]. The OPR was lowest in Asia and appeared to be decreasing over time. Study design, national wealth, and participant age did not explain OPR heterogeneity.

**Conclusion:**

Gender differences in PD prevalence may not be as stark as previously thought. Studies are needed to understand the role of other determinants of gender differences in PD prevalence.

The burden of Parkinson's disease (PD) appears to be increasing and is estimated to reach 13 million PD cases by 2040.^1^ In observational studies, PD tends to affect males more frequently than females; however, the reasons are unclear.^2^, ^3^, ^4^, ^5^ In some parts of the world (eg, South Korea, Japan, and Bolivia), a male predominance of PD is less apparent.^6^, ^7^, ^8^ Variation in prevalence may be explained by case ascertainment (specialist access), social determinants (health beliefs, insurance), or risk factors (ie, genetic, environmental factors, or their interaction).^9^, ^10^, ^11^, ^12^ A study by Pringsheim et al^3^ demonstrated that the male/female ratio for prevalence increased with advancing age and varied by continent (lowest in Asian countries).

In this systematic review and meta‐analysis, we updated our knowledge about gender differences in PD prevalence, building on previous literature.^3^ We hypothesized that study design, economic profile of the country, age at inclusion, or life expectancy could explain gender differences in PD prevalence. Furthermore, as the average age of the global population increased, we expected that there may be differences in the prevalence ratios reported in observational studies.

## Methods

### Data Selection

In this project, we updated a meta‐analysis conducted by Pringsheim et al^3^ between 1985 and 2010. We searched for studies published from 2011 to 2021 with the terms: “Parkinson” and “prevalence”/“epidemiology” on MEDLINE, SCOPUS, and OVID (Table S1). Articles were included if they were population‐based (door‐to‐door surveys and predictive healthcare registry studies using PD diagnostic codes/medication), reported prevalence rates for males and females separately, and data were stratified by age groups. We excluded cohort studies because their design typically captures incidence and case–control studies because of potential selection bias.^13^ Other exclusion criteria were lack of gender/age subgroup analysis, inclusion of secondary parkinsonism, and unavailable full‐text.

Two independent reviewers (A.Z. and S.C.R.) conducted the search and screened abstracts for eligibility. When differentially included, a third reviewer (A.J.N.) was involved. The list of selected prevalence studies was then combined with the studies from Pringsheim et al.^3^ Because of low quality (30), lack of availability of full‐text articles in English (1), and lack of gender subgroups (3) from the list generated by Pringsheim et al,^3^ we could only include 13 of the 47 studies that they included.

### Data Extraction

Extraction of prevalence data was performed by each reviewer including study reference, study design, target population, male/female prevalence, case numbers, total population by gender, and age groups. Age‐standardized or crude prevalence rates were used and converted to cases per 100,000 persons (Supplementary material—Raw OPR data.pdf).

Additional data were extracted from World Health Organization life expectancy (LE) tables—defined as sex‐ and age‐specific death rates at birth for a specific year and region. Therefore, we used LE from the year 2000 for studies published before this year and from the year 2010 for later studies.^14^ The LE gap was calculated as the difference between genders (female LE − male LE). Age at inclusion was extracted from articles either as reported (median) or was calculated from frequency tables (as the rank of the median).^15^ World Bank data was used for the classification of high‐income countries (HIC) and low/middle‐income countries (L/MIC).^16^

### Data Analysis

Prevalence from each study was converted into a male/female ratio: overall male/female prevalence ratio (OPR) with log transformation for normalization. A random‐effects model was used because of high heterogeneity calculated by Cochrane Q statistic and I^2^. Subgroup meta‐analysis and single meta‐regression were used to determine the significance of the different variables: study design (survey vs. predictive), economic country profile, median age at study inclusion, LE gap, and study origin. For study origin, Europe was used as the reference continent. STATA v.19 was used for statistical analysis and figures (Supplementary File—Prevalence MA code).

## Results

**FIG. 1:**
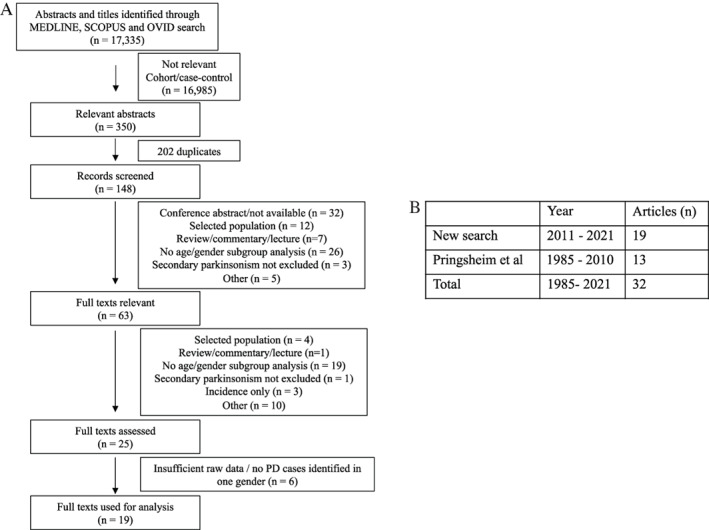
PRISMA flow diagram of article selection and final count of articles selected. (A) Results from search strategy. (B) Final results from the search strategy included 19 articles from 2011 to 2021. These were added to the list of articles from Pringsheim et al,^3^ totaling 32 articles from the years 1985 to 2021

Our search identified 17,335 abstracts (Fig. 1A). Titles and abstracts were screened for relevance and to exclude case–control and cohort studies. From the first round, 350 articles were deemed relevant, with 25 articles included for a full‐text review after further exclusions. An in‐depth review of the articles resulted in the exclusion of another six articles because of insufficient data or no cases of PD identified for one gender (prevalence of 0). The final 19 articles from our search were added to the 13 articles from Pringsheim et al^3^ (Fig. 1B, Table 1, Supplementary File—Supplementary References).

**TABLE 1:** List of included prevalence studies with study characteristics

Author, Year	OPR	Lower CI	Upper CI	Age*	M LE	F LE	F−M survival difference	Age group	Study type	Continent
Orozco, 2020^17^	1.23	1.15	1.32	73	74	80.15	6.15	30+	P	South America
Eusebi, 2019^18^	0.99	0.94	1.05	82	79.47	84.22	4.75	40+	P	Europe
Han, 2019^19^	0.98	0.96	0.99	72	77.12	83.78	6.66	0+	P	Asia
Park, 2019^6^	0.63	0.63	0.64	85	77.12	83.78	6.66	0+	P	Asia
Fleury, 2018^20^	1.45	1.44	1.46	75	80.03	84.18	4.15	0+	P	Europe
Kadastik‐Erme, 2018^21^	1.21	0.99	1.47	77	70.86	80.45	9.59	0+	P	Europe
Marras, 2018^22^	1.37	1.34	1.39	80	78.56	80.8	2.24	45+	P	North America
Valent, 2018^23^	1.28	1.21	1.35	77	79.47	84.22	4.75	0+	P	Europe
Nerius, 2017^24^	1.05	0.99	1.11	77	77.78	82.54	4.76	0+	P	Europe
Khan, 2016^25^	1.77	0.47	6.64	72	61.53	63.9	2.37	50+	S	Asia
Blin, 2015^26^	0.97	0.97	0.98	80	77.96	84.34	6.38	18+	P	Europe
Khedr, 2015^27^	1.39	0.71	2.72	67	67.82	72.69	4.87	0+	S	Africa
Yang, 2015^28^	1.31	0.86	2	70	72.26	77.94	5.68	0+	S	Asia
Zou, 2014^29^	1.67	0.75	3.74	85	72.26	77.94	5.68	70+	P	Asia
El‐Tallawy, 2013^62^	1.32	0.66	2.66	65	67.82	72.69	4.87	40+	S	Africa
Gordon, 2012^30^	1.45	1.34	1.57	70	78.56	80.8	2.24	40+	P	North America
Lökk, 2012^31^	1.19	1.11	1.26	74	79.41	83.15	3.74	0+	P	Europe
Seijo‐Martinez, 2011^32^	2.09	0.75	5.81	80	78.76	84.58	5.82	65+	S	Europe
Osaki, 2011^7^	0.68	0.47	0.99	75	79.46	85.77	6.31	0+	P	Asia
Das, 2008^33^	0.48	0.18	1.28	80	65.69	68.94	3.25	60+	S	India
Barbosa, 2006^34^	1.27	0.68	2.36	82	70.57	77.98	7.41	65+	S	South America
Zhang, 2005^35^	1.17	0.92	1.48	80	72.26	77.94	5.68	55+	S	Asia
Zhang, 2005^35^	1.34	0.88	2.04	63	72.26	77.94	5.68	50+	S	Asia
Bergareche, 2004^36^	0.81	0.31	2.15	80	78.76	84.58	5.82	65+	S	Europe
Tan, 2004^37^	1.55	0.87	2.77	75	79.36	84.01	4.65	50+	S	Asia
Benito‐Leon, 2003^63^	1.58	1.03	2.44	77	78.76	84.58	5.82	65+	S	Europe
Nicoletti, 2003^8^	0.77	0.13	4.58	55	79.47	84.22	4.75	40+	S	South America
Zhang, 2003^38^	1.09	0.67	1.78	80	72.26	77.94	5.68	55+	S	Asia
Kis, 2002^39^	2.86	0.93	8.78	80	79.47	84.22	4.75	60+	S	Europe
Wang, 1996^40^	1.08	0.48	2.45	71.3	69.34	74.15	4.81	50+	S	Asia
Trenkwalder, 1995^41^	3.74	0.73	19.16	78	75.04	80.92	5.88	65+	S	Europe
Wang, 1994^42^	5.66	0.67	48.12	76.5	69.34	74.15	4.81	50+	S	Asia

Abbreviations: OPR, overall male to female prevalence ratio; CI, confidence interval; LE, life expectancy; F, female; M, male; P, predictive; S, survey; WHO, World Health Organization *Age of inclusion is recorded as median. LE values for each gender were extracted from WHO LE at birth from the year 2000 for studies published before this year and from the year 2010 for later studies.

From the pooling effect estimates from the 32 articles in a random‐effects model, we calculated an OPR of 1.18, 95% confidence interval (CI), [1.03, 1.36] with a high degree of heterogeneity (I^2^ = 99.8%) (Figure S1A). There was no evidence of small study bias (*P* = 0.562) (Figure S1B).

**FIG. 2:**
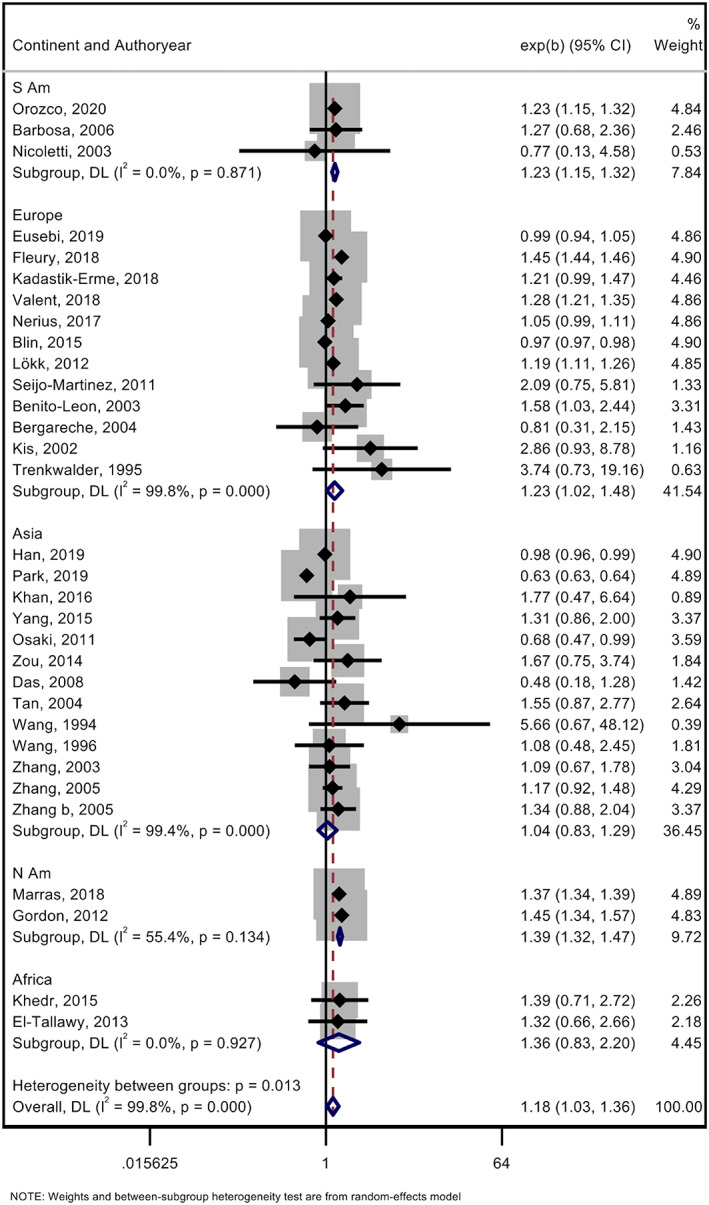
Subgroup meta‐analysis using random effects model for each study continent in the male/female (M/F) prevalence ratio. Random effects meta‐analysis with Cochrane Q statistic of the overall M/F prevalence ratio of each study categorized by its respective continent. The pooled prevalence ratio for all the studies was 1.18, 95% CI, [1.03, 1.36], but heterogeneity was high between groups (I^2^ = 99.8%, *P* = 0.013).

We explored the high heterogeneity observed in the data by considering study design (door‐to‐door survey vs. health records analysis), national economic wealth (HIC vs. L/MIC), and continent. Meta‐regression for study design did not provide evidence that this was a source of heterogeneity (*P* = 0.141). However, studies from L/MIC had a higher OPR of 1.28, 95% CI [1.16, 1.4] compared to studies from HIC of 1.14, 95% CI [0.95, 1.35]. When we looked at OPR between continents, Asia was found to have the smallest OPR, 1.04, 95% CI [0.83, 1.29] (Fig. 2). Meta‐regression for categorical data—economic performance and continent, did not provide evidence that either were sources of heterogeneity (*P* = 0.362 and *P* = 0.100 for Asian compared to European studies, respectively). Similarly, no significant difference was seen between studies from our post‐2011 search and the previous studies included in the analysis (*P* = 0.362).

Next, we investigated whether age at inclusion or LE gap could explain the heterogeneity. The median age for PD patients from the 32 studies was 75.4 (range, 55–85). Meta‐regression using median age did not suggest that this was a cleasource of observed heterogeneity (*P* = 0.059). The LE gap also did not account for observed heterogeneity (*P* = 0.080).

Last, we investigated whether the OPR varied with publication year. A subgroup analysis showed a decreasing trend in the last 30 years: 1990 to 2000 OPR, 2.06; 95% CI [0.72, 5.87], 2000 to 2010 OPR, 1.24; 95% CI [1.06, 1.45] and 2010‐present OPR, 1.15; 95% CI [0.98, 1.35], *P* = 0.019 (Figure S2). However, regression by year of publication was not significant (continuous *P* = 0.158; categorical *P* = 0.311). Multiple regression by publication year and study design was also not significant (*P* = 0.390).

## Discussion

This systematic review and meta‐analysis suggested that the male/female prevalence ratio for PD is lower than has previously been reported by other studies.^2^ The lowest male/female ratio was seen in studies from Asia. We also observed that the OPR may be decreasing over time and may be higher in L/MIC compared with HIC. Such findings may start to shift the paradigm of PD being a “male” dominant disorder, at least in some regions.

Large population‐based studies are best suited for determining the prevalence of diseases like PD.^13^ We included data from healthcare registries and door‐to‐door cross‐sectional studies. Healthcare registries may only cover 50% to 100% of a particular population,^6^, ^24^ meaning that for population‐based prevalence estimates, door‐to‐door studies are considered the gold standard. PD diagnostic criteria vary across regions and institutions, causing potential differences in prevalence estimates for door‐to‐door and healthcare registries.^24^, ^43^ Our analysis did not identify study design as a source of heterogeneity in prevalence ratios, which could be because of our strict inclusion criteria for study design.

There was little evidence to suggest that age at inclusion might explain differences in the gender ratio. Previous studies have suggested that in patients with younger age at onset (AAO), the ratio is closer to 1.^2^ One might expect that genetic factors may have a greater contribution to a lower AAO, although genome‐wide association studies to date have not identified different genetic risk factors for PD between sexes.^12^ Many of these studies over‐represented participants of European ancestry and had lower female inclusion rates.^9^ Efforts to create large trans‐ethnic genomic datasets will help us to better understand PD genetics in under‐represented populations.^44^

A source of heterogeneity that we were unable to explore in‐depth was the role of environmental factors. Rural living and occupational pesticide exposure are associated with an increase in PD risk.^45^, ^46^ Males have traditionally held agricultural occupations, which could explain the higher male PD prevalence.^47^ Increasing urbanization and decreasing organophosphate use in some regions may explain variability in OPRs between continents. Other putative risks/protective factors, including the Mediterranean diet, type 2 diabetes, smoking, and alcohol consumption,^48^, ^49^, ^50^, ^51^, ^52^, ^53^ might contribute to the male predominance of PD.

Healthcare inequalities could account for observed differences in male/female PD prevalence. In L/MIC, such as Benin, 60% of females reported socioeconomic and marital barriers to accessing healthcare.^54^, ^55^ Stigma related to PD symptoms and cultural beliefs about “normal” aging/PD awareness may explain the different levels of healthcare access globally.^56^ Our data suggested that studies from L/MIC have a higher OPR, which may reflect unequal access to healthcare.

Another important consideration is the relationship between disease prevalence, incidence, and survival. Females have a higher AAO and a lower incidence of PD, but also have a longer LE than males.^57^ Female longevity may influence the male/female prevalence imbalance, similar to other neurodegenerative diseases, like Alzheimer's disease.^58^ Our analysis did not support this hypothesis, because LE did not explain heterogeneity in the OPR. However, the LE gap has changed over time,^59^ and this may partly explain why the OPR is also seen to be decreasing over the last 30 years. The recent decrease in the LE gap may be related to environmental exposure, such as increased smoking rates among females^60^ or the increasing integration of females into jobs traditionally held by males. Further studies are needed to understand the effect of LE and environmental/societal factors on PD prevalence.

A limitation of our study was not conducting the search before 2011. We included both door‐to‐door studies—similar to Pringsheim et al,^3^ as well as studies with the newer methodology of predictive algorithms. However, no significant difference was detected between the newly identified studies and those from Pringsheim et al.^3^ Another limitation in our study was the use of both crude and age‐standardized prevalence ratios. A third limitation was classifying the articles by geographic regions rather than individual countries, as genetics, environmental, and demographic factors may vary even between countries from the same region.^61^ This may have impacted our results and further studies of differences within regions are needed.

In conclusion, this systematic review and meta‐analysis showed that the gender ratio for PD prevalence is lower in Asian populations and may have been decreasing in recent years. Further high‐quality epidemiological studies in diverse populations are needed to understand whether the decrease in OPR stems from environmental, societal, and/or genetic factors.

## Author Roles

(1) Research project: A. Conception, B. Organization, C. Execution; (2) Statistical Analysis: A. Design, B. Execution, C. Review and Critique; (3) Manuscript Preparation: A. Writing of the First Draft, B. Review and Critique.

A.Z.: 1A, 1B, 1C, 2A, 2B, 2C, 3A, 3B, 3C.

S.C.R: 1B, 1C, 2C, 3A, 3B.

J.B.: 2A, 2B, 2C, 3B.

R.R.: 3B.

C.M.: 3B.

C.B.: 3B.

I.F.M.: 1C, 3B.

A.J.N.: 1A, 1B, 1C, 2C, 3B.

## Disclosures

**Ethical Compliance Statement:** The authors confirm that the approval of an institutional review board and patient consent was not required for this work. We confirm that we have read the Journal's position on issues involved in ethical publication and affirm that this work is consistent with those guidelines.

**Funding sources and Conflict of Interest:** The Preventive Neurology Unit is funded by the Barts Charity. This work was supported in part by the Intramural Research Program of the National Institute on Aging (NIA). This is present in the disclosures, no conflict but AJN has reported funding sources.

**Financial Disclosures for the previous 12 months:** A.J.N. reports grants from the Barts Charity, Parkinson's United Kingdom, Aligning Science Across Parkinson's, The Michael J. Fox Foundation, and the Virginia Keiley Benefaction. Personal fees/honoraria from Britannia, BIAL, AbbVie, Global Kinetics Corporation, Profile, Biogen, Roche, and UCB are outside of the submitted work. I.F.M. reports grants from Aligning Science Across Parkinson's and The Michael J. Fox Foundation, The Parkinson's Foundation, American Parkinson Disease Association, and National Institutes of Health.

## Supporting information


**Figure S1.** Meta‐funnel plot analyses for reporting bias in prevalence studies. (A) Meta‐analysis using random effects and Cochrane Q statistic for male/female prevalence ratios with an overall prevalence ratio of 1.18, 95% CI, [1.03, 1.36]. (B) Meta‐funnel plot of included studies did not identify any evidence of small studying reporting bias (*P* = 0.562).

**Figure S2.** Subgroup meta‐analysis using random effects model for time‐trends in the M/F prevalence ratio. Random effects meta‐analysis with Cochrane Q statistic of the M/F prevalence ratio of each study categorized by the year of publication. This shows a decreasing trend from 2.06, 95% CI, [0.72, 5.87] in 1990–2000 to 1.24, 95% CI, [1.06, 1.45] in 2000–2010, and to 1.15, 95% CI, [0.98, 1.35] in 2010–2021.

**Table S1.** Search strategies for MEDLINE, SCOPUS, and OVID. Published PD prevalence articles were searched through key terms with the following filters: published between years 1/1/2011–12/30/2021, in English in MEDLINE, SCOPUS, and OVID, sorted by most recent. Abstracts and article information were collated into an excel file, which served as a basis for screening.

Prevalence MA code

Raw OPR data
