# Supplementary figures and images for: Gender Differences in the Prevalence of Parkinson's Disease
Source: Mov Disord Clin Pract. 2022 Nov 14;10(1):86–93. doi: 10.1002/mdc3.13584 (PMC9847309; doi:10.1002/mdc3.13584)

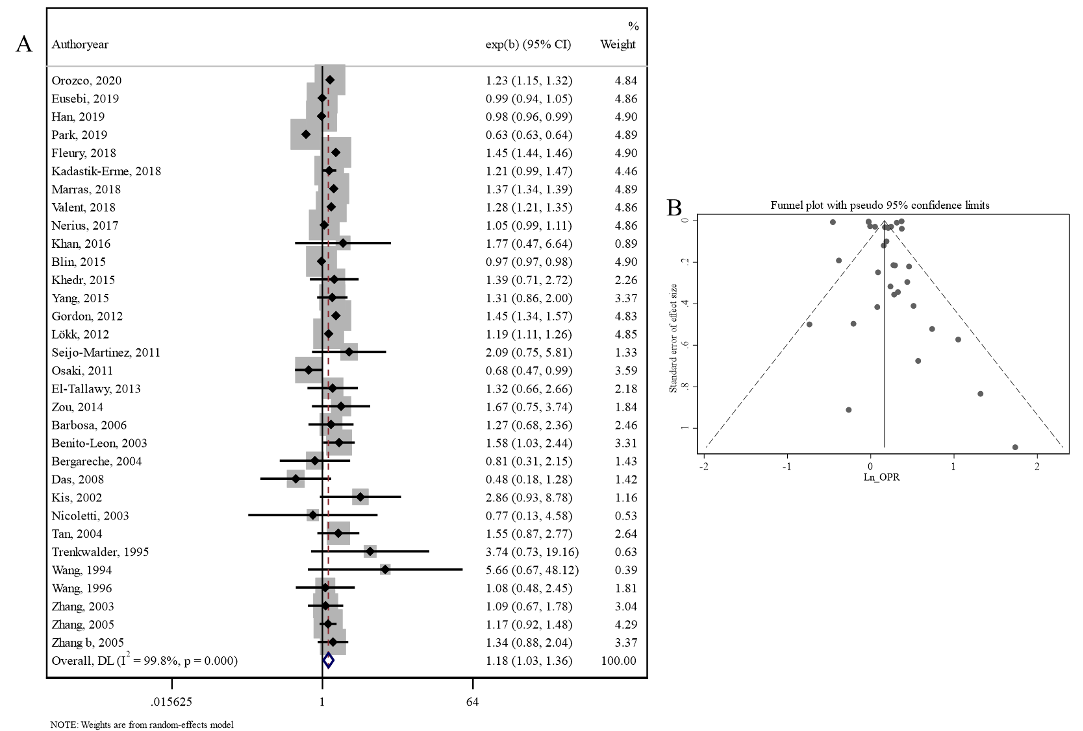

Supplement: Supplementary file 1 — Figure S1. Meta‐funnel plot analyses for reporting bias in prevalence studies. (A) Meta‐analysis using random effects and Cochrane Q statistic for male/female prevalence ratios with an overall prevalence ratio of 1.18, 95% CI, [1.03, 1.36]. (B) Meta‐funnel plot of included studies did not identify any evidence of small studying reporting bias (P = 0.562). [file MDC3-10-86-s003.png]

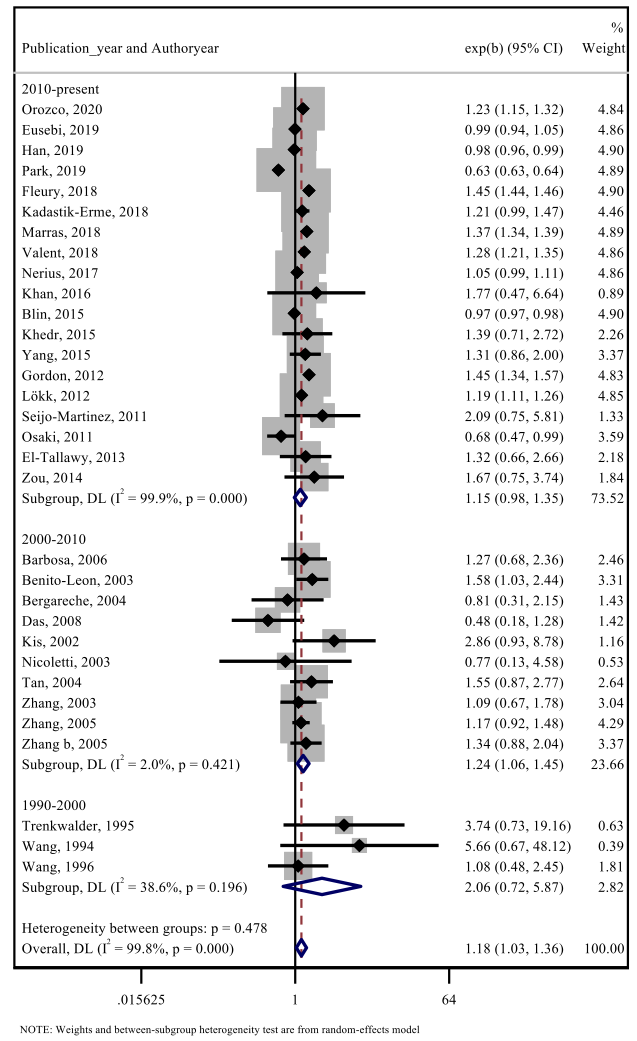

Supplement: Supplementary file 2 — Figure S2. Subgroup meta‐analysis using random effects model for time‐trends in the M/F prevalence ratio. Random effects meta‐analysis with Cochrane Q statistic of the M/F prevalence ratio of each study categorized by the year of publication. This shows a decreasing trend from 2.06, 95% CI, [0.72, 5.87] in 1990–2000 to 1.24, 95% CI, [1.06, 1.45] in 2000–2010, and to 1.15, 95% CI, [0.98, 1.35] in 2010–2021. [file MDC3-10-86-s005.png]
