# Supplementary material for: Gender Differences in the Prevalence of Parkinson's Disease
Source: Mov Disord Clin Pract. 2022 Nov 14;10(1):86–93. doi: 10.1002/mdc3.13584 (PMC9847309; doi:10.1002/mdc3.13584)
Supplement: Supplementary file 3 — Table S1. Search strategies for MEDLINE, SCOPUS, and OVID. Published PD prevalence articles were searched through key terms with the following filters: published between years 1/1/2011–12/30/2021, in English in MEDLINE, SCOPUS, and OVID, sorted by most recent. Abstracts and article information were collated into an excel file, which served as a basis for screening. [file MDC3-10-86-s004.docx]

| DATABASE | SEARCH STRATEGY |
| --- | --- |
| MEDLINE | "Parkinson disease"[All Fields] AND "prevalence"[All Fields]; Filters: Journal Article, English, MEDLINE, from 2011/1/1 - 2021/12/30 Sort by: Most Recent |
|  | "Parkinson disease"[All Fields] AND "epidemiology"[All Fields]; Filters: Journal Article, English, MEDLINE, from 2011/1/1 - 2021/12/30 Sort by: Most Recent |
| SCOPUS | ( TITLE-ABS-KEY ( {Parkinson disease} AND {prevalence} ) AND LANGUAGE ( English ) ) AND PUBYEAR > 2011 |
|  | ( TITLE-ABS-KEY ( {Parkinson disease} AND {epidemiology} ) AND LANGUAGE ( English ) ) AND PUBYEAR > 2011 |
| OVID | "Parkinson disease" AND "prevalence" {Including Limited Related Terms}; Ovid, 2011 to current, English |
|  | "Parkinson disease" AND "epidemiology" {Including Limited Related Terms}; Ovid, 2011 to current, English |
