# Supplementary material for: Gender Differences in the Prevalence of Parkinson's Disease
Source: Mov Disord Clin Pract. 2022 Nov 14;10(1):86–93. doi: 10.1002/mdc3.13584 (PMC9847309; doi:10.1002/mdc3.13584)
Supplement: Supplementary file 4 — Prevalence MA code [file MDC3-10-86-s002.docx]

Code as used in the article:

#Install functions:

ssc install metan

ssc install metareg

ssc install metabias

ssc install metafunnel

#Set working directory and import csv file:

cd "*H:\Gender MA*"

import delimited "*H:\Gender MA\Data\Raw OPR data.csv*"

#Run MA:

meta set ln_opr ln_lower_ci_95 ln_upper_ci_95, studylabel(authoryear) random civartolerance(0.01)

metan ln_opr ln_lower_ci_95 ln_upper_ci_95, random eform label(namevar= authoryear)

#Run publication bias analysis:

metabias ln_opr _seES, egger

metafunnel ln_opr _seES

#Run meta-regression for binary variables - study type:

xi: metareg ln_opr i.study_type, wsse(_seES)

#Run subgroup meta-analysis - economic performance of country:

metan ln_opr ln_lower_ci_95 ln_upper_ci_95, random eform label(namevar= authoryear) by( country_reclass )

#Run subgroup meta-analysis - continent:

metan ln_opr ln_lower_ci_95 ln_upper_ci_95, random eform label(namevar= authoryear) by( continent )

#Run meta-regression for binary variables - continent:

gen continent2=1 if continent=="Europe"

replace continent2=2 if continent=="Asia"

replace continent2=3 if continent=="N Am"

replace continent2=4 if continent=="S Am"

replace continent2=5 if continent=="Africa"

xi: metareg ln_opr i.continent2, wsse(_seES )

#Run meta-regression for binary variables – articles from the new search versus articles from old search (Pringsheim):

xi: metareg ln_opr i.update, wsse(_seES)

#Run meta-regression for continuous variables - median age:

xi: metareg ln_opr median_age , wsse(_seES )

#Run meta-regression for continuous variables - life expectancy difference between females and males:

xi: metareg ln_opr f_to_m_difference_in_life_expect, wsse(_seES )

#Run subgroup meta-analysis - year of publication:

metan ln_opr ln_lower_ci_95 ln_upper_ci_95, random eform label(namevar= authoryear) by( publication_year)
