# Supplementary material for: Gender Differences in the Prevalence of Parkinson's Disease
Source: Mov Disord Clin Pract. 2022 Nov 14;10(1):86–93. doi: 10.1002/mdc3.13584 (PMC9847309; doi:10.1002/mdc3.13584)
Supplement: Supplementary file 5 — Raw OPR data [file MDC3-10-86-s001.pdf]

Author	Year	Authoryear	Prevalence_M	PD_M	Total_M	NonPD_M	Prevalence_F	PD_F	Total_F	NonPD_F	Total_cases	Total_pop	OPR	Ln_OPR	Lower_CI_95	Upper_CI_95	Ln_lower_CI_95	Ln_upper_CI_95	Age_group	Country_reclass	Continent	Study_type	Median_age	Life_expectancy_M	Life_expectancy_F	F_to_M_difference_in_Life_expectancy	Publication_year	Update
Orozco	2020	Orozco, 2020	175	1750	1000000	998250	141.91	1514	1066873	1065359	3264	2066873	1.2332	0.2096	1.1513	1.3209	0.1409	0.2783	0	2	S Am	2	73	74	80.15	6.15	2010-present	1
Eusebi	2019	Eusebi, 2019	540	2628	486667	484039	544	2848	523529	520681	5476	1010196	0.9926	-0.0074	0.9415	1.0465	-0.0603	0.0455	2	1	Europe	2	82	79.47	84.22	4.75	2010-present	1
Han	2019	Han, 2019	113.1	38157	33737401	33699244	116	54236	46755172	46700936	92393	80492573	0.975	-0.0253	0.9623	0.9878	-0.0384	-0.0122	0	1	Asia	2	72	77.12	83.78	6.66	2010-present	1
Park	2019	Park, 2019	108.4	32002	29522140	29490138	170.8	48745	28539227	28490482	80747	58061367	0.6347	-0.4547	0.6258	0.6437	-0.4688	-0.4406	0	1	Asia	2	85	77.12	83.78	6.66	2010-present	1
Fleury	2018	Fleury, 2018	223.7	227697	101786768	101559071	154.5	242815	157161812	156918997	470512	258948580	1.4479	0.3701	1.4396	1.4562	0.3644	0.3758	0	1	Europe	2	75	80.03	84.18	4.15	2010-present	1
Kadastik-Erme	2018	Kadastik-Erme, 2018	355	160	45070	44910	294	271	92177	91906	431	137247	1.2075	0.1885	0.9935	1.4676	-0.0065	0.3836	0	1	Europe	2	77	70.86	80.45	9.59	2010-present	1
Marras	2018	Marras, 2018	667	20366	3053373	3033007	488	23491	4813730	4790239	43857	7867103	1.3668	0.3125	1.3415	1.3926	0.2938	0.3312	3	1	N Am	2	80	78.56	80.8	2.24	2010-present	1
Valent	2018	Valent, 2018	747.08	2270	303850	301580	585.33	2465	421130	418665	4735	724980	1.2763	0.244	1.2058	1.351	0.1872	0.3008	0	1	Europe	2	77	79.47	84.22	4.75	2010-present	1
Nerius	2017	Nerius, 2017	820	2002	244146	242144	779	2734	350963	348229	4736	595109	1.0526	0.0513	0.9939	1.1148	-0.0061	0.1087	0	1	Europe	2	77	77.78	82.54	4.76	2010-present	1
Khan	2016	Khan, 2016	530	8	1509	1501	300	3	1000	997	11	2509	1.7667	0.5691	0.4698	6.6433	-0.7554	1.8936	4	2	Asia	1	72	61.53	63.9	2.37	2010-present	1
Blin	2015	Blin, 2015	304	95716	31485526	31389810	312	104557	33511859	33407302	200273	64997385	0.9744	-0.026	0.9659	0.9829	-0.0347	-0.0172	1	1	Europe	2	80	77.96	84.34	6.38	2010-present	1
Khedr	2015	Khedr, 2015	503	21	4175	4154	363	14	3857	3843	35	8032	1.3857	0.3262	0.7056	2.7211	-0.3487	1.001	0	2	Africa	1	67	67.82	72.69	4.87	2010-present	1
Yang	2015	Yang, 2015	1680	51	3036	2985	1280	37	2891	2854	88	5926	1.3125	0.2719	0.8622	1.998	-0.1483	0.6921	0	2	Asia	1	70	72.26	77.94	5.68	2010-present	1
Zou	2014	Zou, 2014	2440	219	8975	8756	1460	6	411	405	225	9386	1.6712	0.5136	0.7472	3.7381	-0.2914	1.3186	8	1	Asia	2	85	72.26	77.94	5.68	2010-present	1
El-Tallawy	2013	El-Tallawy, 2013	240.38	20	8320	8300	181.51	13	7162	7149	33	15482	1.3243	0.2809	0.6593	2.6604	-0.4166	0.9785	2	1	Africa	1	65	67.82	72.69	4.87	2010-present	1
Gordon	2012	Gordon, 2012	1012	1322	130632	129310	697.2	1291	185169	183878	2613	315802	1.4515	0.3726	1.3448	1.5667	0.2962	0.449	2	2	N Am	2	70	78.56	80.8	2.24	2010-present	1
Lökk	2012	Lökk, 2012	213.18	2127	997748	995621	179.74	1836	1021475	1019639	3963	2019224	1.186	0.1706	1.1143	1.2624	0.1082	0.233	0	2	Europe	2	74	79.41	83.15	3.74	2010-present	1
Seijo-Martinez	2011	Seijo-Martinez, 2011	2860	9	315	306	1370	6	438	432	15	753	2.0876	0.736	0.7507	5.8056	-0.2868	1.7588	7	1	Europe	1	80	78.76	84.58	5.82	2010-present	1
Osaki	2011	Osaki, 2011	88	43	48864	48821	129	73	56589	56516	116	105453	0.6822	-0.3825	0.4681	0.9941	-0.7591	-0.0059	0	1	Asia	2	75	79.46	85.77	6.31	2010-present	1
Das	2008	Das, 2008	216	6	2772	2766	451	12	2658	2646	18	5430	0.4789	-0.7362	0.1799	1.2753	-1.7156	0.2432	6	2	Asia	1	80	65.69	68.94	3.25	2000-2010	0
Barbosa	2006	Barbosa, 2006	3800	17	447	430	3000	22	733	711	39	1181	1.2667	0.2364	0.6801	2.3591	-0.3855	0.8583	7	2	S Am	1	82	70.57	77.98	7.41	2000-2010	0
Zhang	2005	Zhang, 2005	1040	130	12500	12370	890	147	16517	16370	277	29017	1.1685	0.1558	0.924	1.4778	-0.0791	0.3906	5	1	Asia	1	80	72.26	77.94	5.68	2000-2010	0
Zhang b	2005	Zhang b, 2005	614	41	6678	6637	459	45	9804	9759	86	16481	1.3377	0.2909	0.8772	2.04	-0.1311	0.713	4	2	Asia	1	63	72.26	77.94	5.68	2000-2010	0
Bergareche	2004	Bergareche, 2004	1300	6	462	456	1600	12	750	738	18	1212	0.8125	-0.2076	0.307	2.15	-1.1808	0.7655	7	2	Europe	1	80	78.76	84.58	5.82	2000-2010	0
Tan	2004	Tan, 2004	310	25	8065	8040	200	21	10500	10479	46	18565	1.55	0.4383	0.8683	2.7668	-0.1412	1.0177	4	1	Asia	1	75	79.36	84.01	4.65	2000-2010	0
Benito-Leon	2003	Benito-Leon, 2003	1900	43	2263	2220	1200	38	3167	3129	81	5430	1.5833	0.4595	1.0268	2.4414	0.0265	0.8926	7	2	Europe	1	77	78.76	84.58	5.82	2000-2010	0
Nicoletti	2003	Nicoletti, 2003	248	2	806	804	323	3	929	926	5	1735	0.7678	-0.2642	0.1286	4.5837	-2.051	1.5225	2	2	S Am	1	55	79.47	84.22	4.75	2000-2010	0
Zhang	2003	Zhang, 2003	1200	30	2500	2470	1100	34	3091	3057	64	5591	1.0909	0.087	0.6696	1.7774	-0.4011	0.5751	5	2	Asia	1	80	72.26	77.94	5.68	2000-2010	0
Kis	2002	Kis, 2002	4800	6	336	330	1680	6	414	408	12	750	2.8571	1.0498	0.93	8.7779	-0.0726	2.1722	6	1	Europe	1	80	79.47	84.22	4.75	2000-2010	0
Wang	1996	Wang, 1996	610	12	1967	1955	564	11	1950	1939	23	3918	1.0816	0.0784	0.4784	2.4453	-0.7373	0.8942	4	1	Asia	1	71	69.34	74.15	4.81	1990-2000	0
Trenkwalder	1995	Trenkwalder, 1995	1270	5	394	389	340	2	588	586	7	982	3.7353	1.3178	0.7283	19.1577	-0.3171	2.9527	7	1	Europe	1	78	75.04	80.92	5.88	1990-2000	0
Wang	1994	Wang, 1994	2212.39	5	226	221	390.63	1	256	255	6	482	5.6637	1.7341	0.6666	48.1195	-0.4055	3.8737	4	2	Asia	1	77	69.34	74.15	4.81	1990-2000	0

Legend

Header	Explanation									
Author	First author									
Year	Year of publication									
Authoryear										
Prevalence_M	Reported male prevalence / 100,000 persons									
PD_M	Number of male PD cases									
Total_M	Total number of males in population									
NonPD_M	Number of males without PD									
Prevalence_F	Reported female prevalence / 100,000 persons									
PD_F	Number of female PD cases									
Total_F	Total number of females in population									
NonPD_F	Number of females without PD									
Total_cases	Total number of PD cases in population									
Total_pop	Total population									
OPR	Overall prevalence rate (Male-to-Female)									
Ln_OPR	Natural logarithm of OPR									
Lower_CI_95	95% lower CI									
Upper_CI_95	95% upper CI									
Ln_lower_CI_95	Natural logarithm of 95% lower CI									
Ln_upper_CI_95	Natural logarithm of 95% upper CI									
Age_group	Ages groups included in study	0 - all ages	1 - 18+	2 - 40+	3 - 45+	4 - 50+	5 - 55+	6 - 60+	7 - 65+	8 - 70+
Country_reclass	Economic profile of country	1 - HIC	2 - L/MIC							
Continent	Study continent									
Study_type	Study design	1 - Door-to-door	2 - Predictive							
Median_age	Median age at inclusion									
Life_expectancy_M	Male life expectancy at birth based on WHO data									
Life_expectancy_F	Female life expectancy at birth based on WHO data									
F_to_M_difference_	Difference between female and male life expectancy									
Publication_year	Year of publication	1990-2000	2000-2010	2010-present						
Update	Article from new/old search	1 - New search	0 - Pringsheim *et al*							
